# Expanding the Use of Peroxygenase from Oat Flour in Organic Synthesis: Enantioselective Oxidation of Sulfides

**DOI:** 10.3390/ijms24087464

**Published:** 2023-04-18

**Authors:** Claudia Sanfilippo, Federica Cernuto, Angela Patti

**Affiliations:** CNR—Istituto di Chimica Biomolecolare, Via Paolo Gaifami 18, I-95126 Catania, Italy

**Keywords:** peroxygenase, sulfides, chiral sulfoxides, oat flour

## Abstract

Biocatalyzed oxidations are an important target in sustainable synthesis since chemical oxidations often require harsh conditions and metal-based catalysts. A raw peroxygenase-containing enzymatic preparation from oat flour was tested as a biocatalyst for the enantioselective oxidation of sulfides to sulfoxides and the variations of some reaction parameters were evaluated. Under optimal conditions, thioanisole was fully converted into the corresponding (*R*)-sulfoxide with high optical purity (80% *ee*) and the same stereopreference was maintained in the oxidation of some other sulfides. Changes in the substituent on the sulfur atom affected the selectivity of the enzyme and the best results were obtained with phenyl methoxymethyl sulfide, which gave the corresponding sulfoxide in 92% *ee* as exclusive product. The over-oxidation of sulfides to sulfones was instead detected in all the other cases and preferential oxidation of the (*S*)-enantiomer of the sulfoxide intermediate was observed, albeit with low selectivity. Carrying out the oxidation of thioanisole up to the 29% formation of sulfone led to enhancement of the sulfoxide optical purity (89% *ee*). The activity in sulfoxidation reactions, in addition to that reported in the epoxidation of different substrates, makes this plant peroxygenase a promising and useful tool in organic synthesis.

## 1. Introduction

Chiral sulfoxides are an important class of organosulfur compounds with relevant applications as intermediates in asymmetric synthesis as well as in medicinal chemistry. Since the early 1980′s, they have become very popular as chiral auxiliaries [1,2] due to their configurational stability and easy removal in mild conditions. The high asymmetric induction exerted by the chiral sulfinyl group has been related to steric and stereoelectronic differences between the substituents at the sulfur atom and has been ascertained in a variety of nucleophilic substitutions, metal-promoted reactions and, more recently, C-H functionalization [3,4]. 

Enantiomerically pure sulfoxides have been used in asymmetric synthesis [5] as effective organocatalysts [6,7,8], additives or Lewis bases [9,10] and several metal complexes incorporating simple or bidentate sulfoxides as ligands have been developed [11].

Sulfur chirality is recognized to play an important role also in biological processes [12] and sulfoxide functional group is a valuable scaffold in the field of medicinal chemistry [13], being part of a variety of approved drugs and clinical candidates. Among these, in the family of proton pump inhibitors [14,15] omeprazole was the best-selling drug in the treatment of gastric acid hypersecretion, but the superior clinical efficacy (improved bioavailability, acid control, healing and symptoms resolution) proved for its (*S*)-enantiomer esomeprazole [16,17] led to a growing market for this drug compared to the original formulation containing the racemic molecule. More recently, dexlansoprazole, the (*R*)-enantiomer of lansoprazole, was launched on the market in a dual delayed-release formulation able to maintain a more prolonged drug exposure in human plasma compared to the parent racemic drug [18].

The synthesis of chiral sulfoxides mainly relies on catalytic asymmetric oxidation of prochiral sulfides with benign oxidants, as *tert*-butyl hydroperoxide (TBHP) or H_2_O_2_, in the presence of metal-chiral ligand complexes or organocatalysts and an impressive variety of effective systems has been developed. This reaction is still considered the most general and convenient approach to the synthesis of optically active sulfoxides, although alternative strategies based on the enantioselective formation of C-S bond or kinetic resolution of racemic sulfoxides, have been also explored [19,20,21,22]. 

Biocatalysis offers a valuable alternative to chemical methods for the inherent stereoselectivity of the enzymes, whose available portfolio is continuously growing thanks to the modern protein-engineering techniques. Most of the biotransformations aimed to the synthesis of chiral sulfoxides exploit oxidative enzymes such as cytochromes P450- and Bayer Villiger-monooxygenases in microbial or fungal whole cell systems, which also encompass the cofactor-recycling enzymes, or in purified form [23,24]. Depending on the starting sulfide, good to excellent optical purities (70–99% *ee*) of the target sulfoxides have been reported in many cases, with a striking example in preparation of esomeprazole (99.9% *ee*), optimized at pilot-scale levels by using an engineered cyclohexanone monooxygenase from *Acinetobacter calcoaceticus* and molecular oxygen as oxidant, in the presence of a formate dehydrogenase for NADPH regeneration [25].

Peroxygenases and peroxidases constitute another group of oxidative enzymes that use hydroperoxides as oxidants and display high potential in the oxyfunctionalization of organic molecules [26,27,28] without the need of cofactors, and some of them were found able to enantioselectively oxidize sulfur atom of sulfides [29,30] even in a non-conventional medium [31,32,33]. Among these enzymes, unspecific peroxygenases (UPOs, E.C 1.11.2.1), which are extracellular enzymes produced by many fungi, proved to be versatile catalysts in different reactions, also including the functionalization of non-activated carbons and heteroatoms [34]. In plants, most interest has been focused on peroxygenase from oat seeds (E.C. 1.11.2.3), which is involved in the oxidative metabolism of fatty acids [35] and has been characterized at molecular [36] and genetic [37] level. The microsome fraction from oat seeds was found active in catalyzing the epoxidation of linoleic acid [38] and the same reaction was effectively carried out on some alkenes and polyunsaturated fatty acids [39] by using a membrane-bound enzymatic preparation with minimal prior purification. However, data on the optical purity of the obtained epoxides were not reported. 

In our previous work, we have shown that the peroxygenase activity is maintained in a lyophilized raw preparation from oat seeds and applied it in the selective epoxidation of EPA and DHA (and the corresponding ethanolamides), also assigning the absolute configuration and enantiomeric excess of the obtained products [40,41]. The same enzymatic preparation catalyzed the epoxidation of both enantiomers of limonene with excellent regio- and diastereoselectivity [42]. As a part of a study aimed to better explore the application of oat peroxygenase in asymmetric organic synthesis, the oxidation of thioanisole and some related sulfides was investigated and the obtained results are reported here.

## 2. Results

### 2.1. Peroxygenase-Catalyzed Oxidation of Sulfides to Sulfoxides

**Scheme 1:**
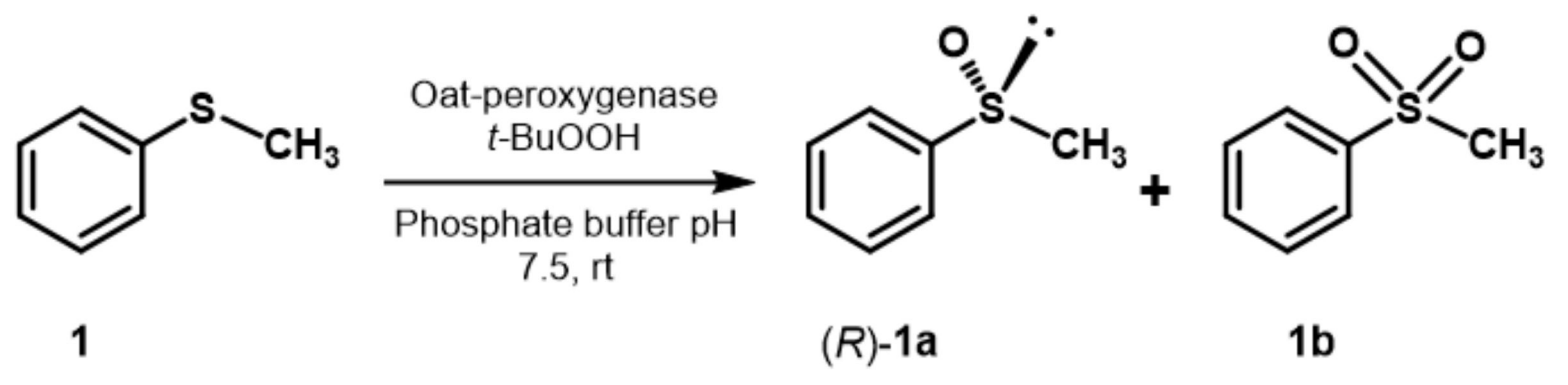
Oxidation of thioanisole catalyzed by peroxygenase from oat flour.

At the onset of our work, the standard conditions developed in the peroxygenase-catalyzed epoxidation of different substrates [40,42] were applied for the oxidation of thioanisole **1** (Scheme 1). The peroxygenase biocatalyst was obtained by lyophilization of the aqueous extract of oat flour, as previously described [40], and the slight excess (1.1 eqv.) of *tert*-butyl hydroperoxide (TBHP) oxidant was added in multiple portions over 1 h to avoid enzyme deactivation. In this time, substrate **1** was fully converted into sulfoxide (*R*)-**1a** and a little amount (4%) of sulfone **1b** was also formed (Scheme 1). 

The sulfoxide **1a** was formed in good optical purity (76% enantiomeric excess, *ee*) and its *R*-absolute configuration was assigned based on elution order of the enantiomer peaks in chiral HPLC analysis [43]. The observed stereopreference was found in agreement with that reported for other peroxygenases from a microbial source [29,30]. Control experiments confirmed that the reaction is enzyme catalyzed, as the spontaneous oxidation of **1** with TBHP was not observed within 12 h.

**Figure 1:**
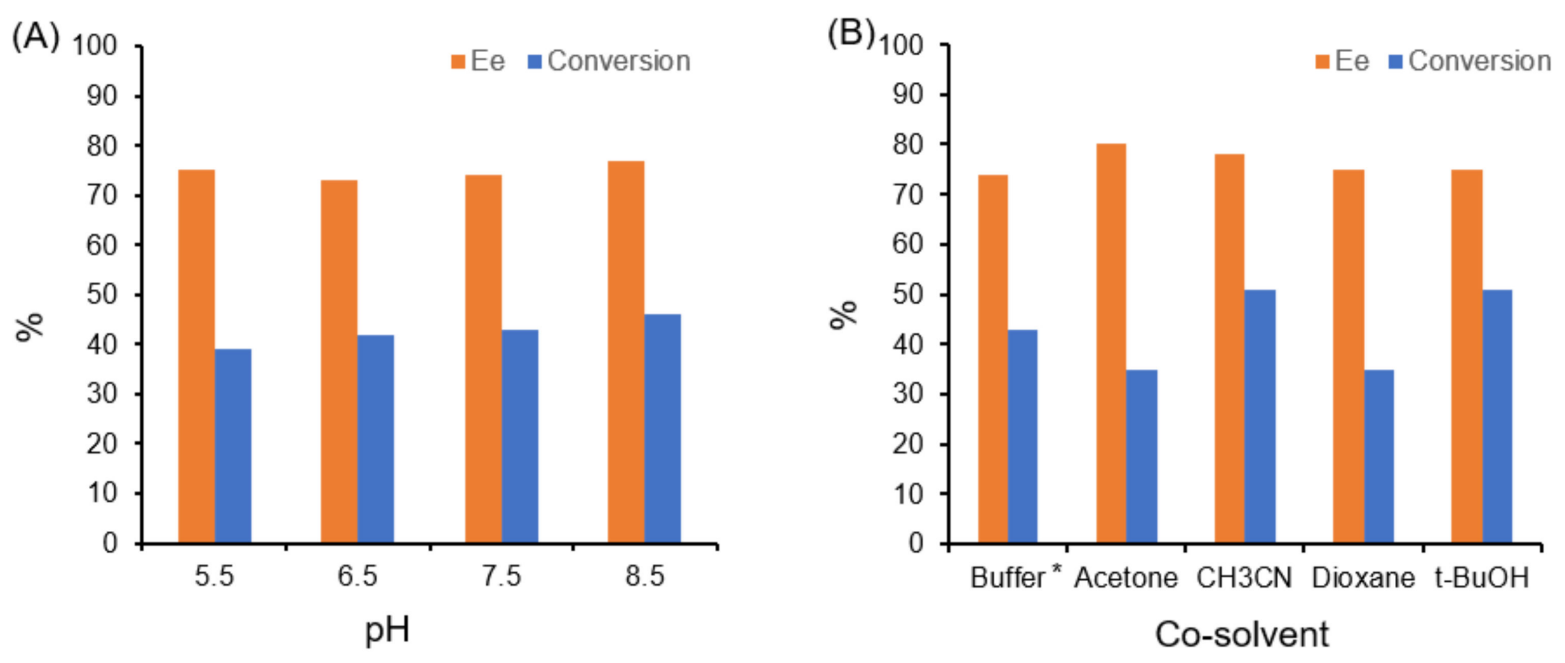
Influence of pH variation (**A**) and addition of cosolvents (20% *v*/*v*) in the reaction medium (**B**) for the biocatalyzed sulfoxidation of thioanisole (1 h reaction time, 0.6 eqv. of oxidant added, Buffer * = pH 7.5).

To evaluate the selectivity of the enzyme in different experimental conditions, a series of oxidation reactions were then carried out on **1** providing to use a sub-stoichiometric amount of TBHP to avoid the formation of **1b**, which in principle could affect the optical purity of **1a** through a preferential over-oxidation of one of its enantiomers. Changes of buffer pH in the range 5.5–8.5 did not result in significant increase in enzymatic activity in both reaction rate and enantioselectivity (Figure 1A). Different organic co-solvents in the reaction medium were well tolerated by oat peroxygenase and a slight increase in optical purity of (*R*)-**1a** was observed in the presence of CH_3_CN or acetone (20% *v*/*v*) compared to a pure buffer (Figure 1B). Increasing concentration of acetone up to 50% *v*/*v* led to a higher reaction rate without further improving the selectivity and no significant changes were obtained, even by varying the reaction temperature in the range of 5–35 °C.

Some aromatic sulfides other than thioanisole were then subjected to biocatalyzed oxidation in the presence of 20% acetone as co-solvent and buffer at pH 7.5, chosen as standard conditions (Table 1), and the reactions were monitored until all the substrates were not detected anymore. All the substrates were fully converted within 1–3 h and the highest reaction rate was observed for benzyl methyl sulfide **5** which, however, gave the corresponding sulfoxide **5a** with the lowest enantioselectivity. Changing the methyl group on sulfur atom with an ethyl group led to sulfoxide **2a** with moderate optical purity (58% *ee*), while the introduction of a methoxymethyl group positively affected the enzyme selectivity resulting in the formation of sulfoxide **3a** in 90% *ee*. In a further refinement of this reaction, using pure buffer pH 7.5 as solvent, compound **3a** was obtained in 92% *ee*. 

The oxidation of *p*-methylphenyl methyl sulfide **4** proceeded with low selectivity, unlike what was reported for the reactions with UPO and CPO peroxygenases [29,30], which were found to be more selective with this substrate compared to thioanisole. 

Although a reversed chirality descriptor is applied to **3a** from the priority rules, all the obtained sulfoxides possess the same spatial orientation of the substituents on sulfur atom, resulting from the stereopreference of peroxygenase in the S-O bond formation step. Over-oxidation was observed for all sulfides, except **3**, and low amounts (6–12%) of sulfones were detected at the end of reaction without significant increase over 24 h, maybe due to enzyme deactivation. 

For preparative purposes, the biocatalyzed oxidation of **1** was scaled up to 75 mM and suitably quenched when the substrate was fully converted to give (*R*)-**1a** in 96% yield and 80% *ee*. In a parallel reaction carried out in the optimal conditions for the formation of sulfone **1b** (see infra), the sulfoxide (*R*)-**1a** was obtained in 64% isolated yield and 89% *ee*. Oxidation of **3** in preparative scale, instead, afforded (*S*)-**3a** as exclusive product, which was isolated in excellent yield and optical purity (90%, 92% *ee*).

Considering that we used a raw enzyme preparation, a possible contribution of different oxidative enzymes to the observed stereoselectivity could not be ruled out and some experiments aimed to better clarify this issue were performed.

The biocatalytic oxidation in hand was strictly hydroperoxide-dependent and, indeed, in the presence of sole oxygen as oxidant the starting sulfides were recovered unchanged. Among the enzymes able to use hydroperoxide as oxidant, cytochromes or monooxygenases were considered not active in our reaction conditions for the lack of any added ancillary cofactor, essential for their activity. 

A specific assay for peroxidase activity (guaiacol oxidation) gave a negative result for our preparation and the oxidation of **1** was not affected by the addition of 2,2,5,7,8-pentamethyl-6-chromanol (a water-soluble chain-breaking antioxidant), suggesting an oxygen transfer mechanism (peroxygenase activity) rather than a radical one (peroxidase activity) for the reaction. 

Finally, parallel reactions were carried out on **1** using the lyophilized raw enzyme preparation and the corresponding microsome fraction (100,000 g pellet), in which oat peroxygenase is known to be localized [36]. Sulfoxide (*R*)-**2** was obtained with the same optical purity from both reactions, indicating that soluble enzymes or proteins (such as albumins) possibly extracted from the seeds of oat did not interfere with the stereochemical outcome of sulfoxidation. Based on these considerations, we assumed that the active enzyme in our oat preparation is a peroxygenase. 

Since a non-negligible enzymatic activity was lost during the sub-cellular fractionation, the use of a raw enzymatic preparation offers a valuable alternative in preparative synthesis, while also avoiding time-consuming and ultracentrifugation-based purification of oat peroxygenase.

### 2.2. Over-Oxidation of Sulfoxides to Sulfones

The formation of sulfones observed during the oat peroxygenase-catalyzed oxidation of sulfides prompted us to better investigate this over-oxidation reaction, with the aim to gain a better insight into the enzyme features and evaluate the possibility to enhance the optical purity of a target sulfoxide through preferential oxidation of its minor enantiomer to the corresponding sulfone (kinetic amplification). Sulfone formation has not been reported for CPO- and UPO-catalyzed sulfoxidation reactions, while it is rather common with bacterial monooxygenases, which have indeed been exploited for kinetic resolution of sulfoxides [44,45,46,47]. More recently, biocatalyzed resolution of racemic sulfoxides has been reported with reductive enzymes as dimethyl sulfoxide reductase [48,49] or methionine sulfoxide reductase in whole cell systems [50,51] or in purified form [52,53]. 

When the standard peroxygenase-catalyzed oxidation of thioanisole was carried out by using 2 eqv. of TBHP oxidant, sulfone **1b** began to be detected after all the substrate **1** was converted into **1a** and chiral HPLC analysis of the reaction mixture at different times revealed that the optical purity of the formed (*R*)-**1a** improved slightly with an increasing amount of **1b**, suggesting the occurrence of some selective recognition of the (*S*)-**1a** in the over-oxidation step. In the reaction in hand, the *ee* of **1a** varied from 80% at the early stage of the reaction up to 85% at the end of the reaction, when 22% of **1b** was present in the mixture and did not increase further. 

**Scheme 2:**
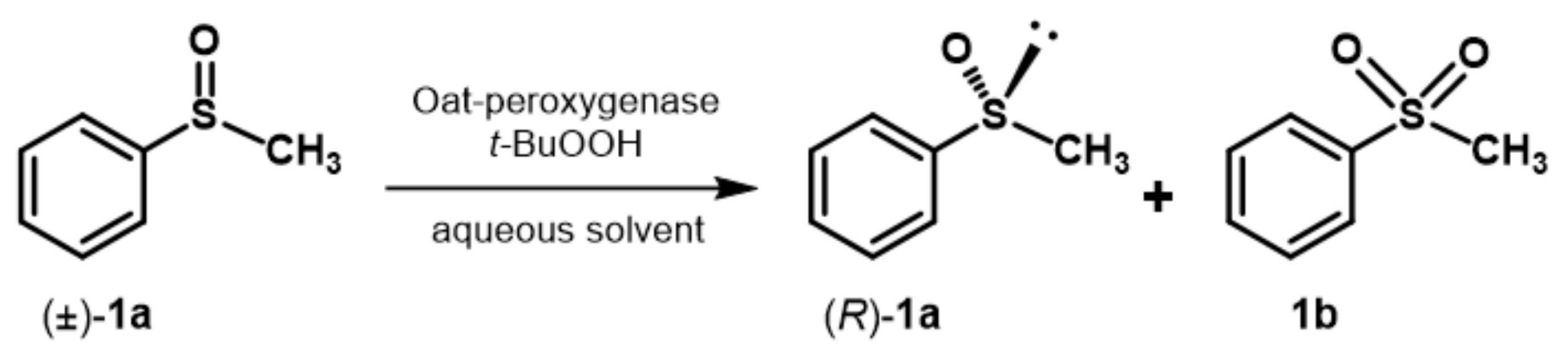
Kinetic resolution of phenyl methyl sulfoxide catalyzed by peroxygenase from oat flour.

A set of reactions was then carried out, starting from (±)-**1a,** with the aim to determine the enantioselectivity E of kinetic resolution [54] in different experimental conditions and it was confirmed that the (*S*)-enantiomer of substrate is preferentially oxidized to sulfone **1b** (Scheme 2).

**Figure 2:**
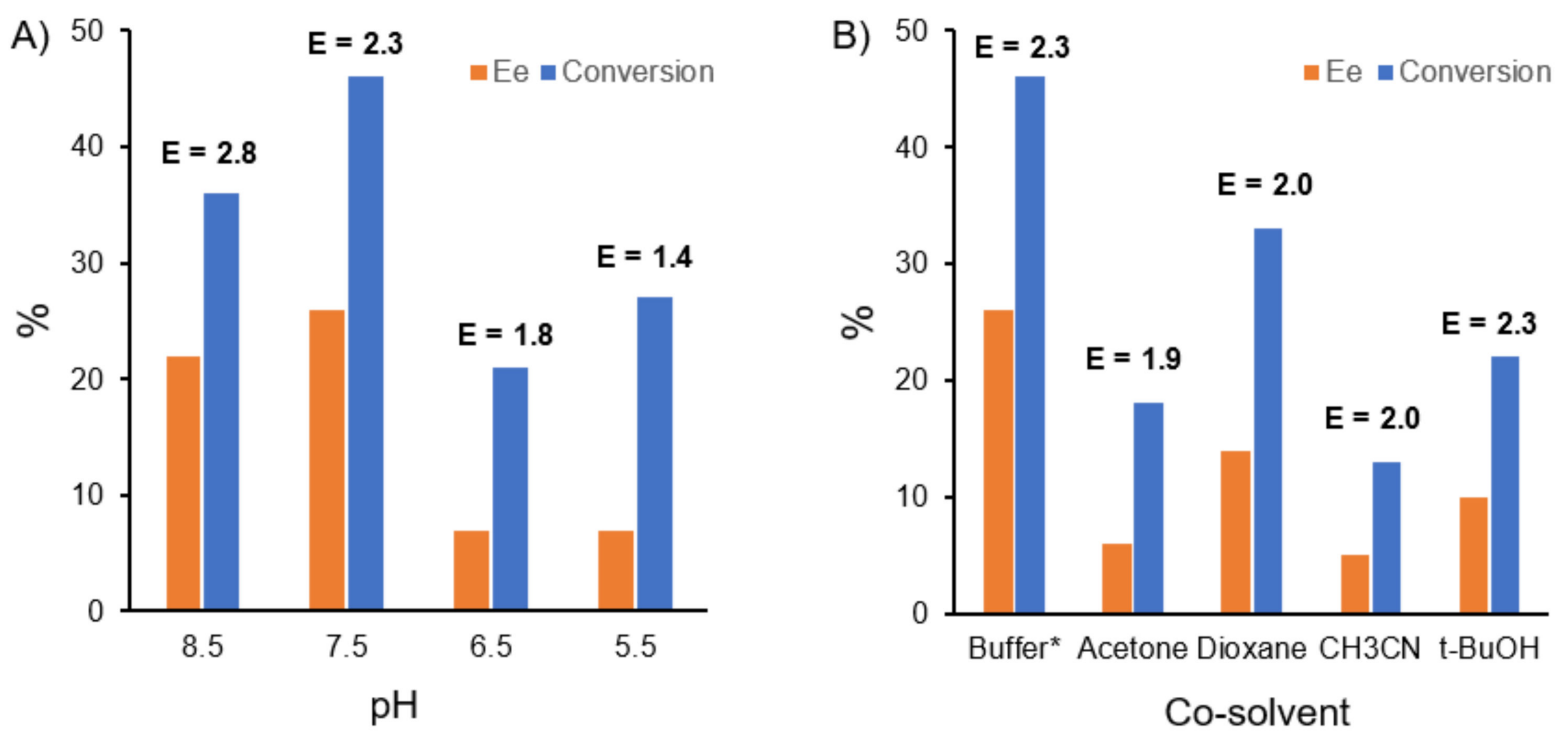
Influence of pH variation (**A**) and addition of co-solvents (20% *v*/*v*) in the reaction medium (**B**) for the biocatalyzed kinetic resolution of methyl phenyl sulfoxide (1 h reaction time, 0.5 eqv. of oxidant, Buffer * = pH 7.5). E values calculated according to [54].

Compared to the oxidation of **1** to **1a**, the biocatalyzed formation of **1b** from (±)-**1a** appeared to be more influenced in the reaction rate by the changes in buffer pH or the nature of the co-solvent, but in all the cases the observed enantioselectivity was disappointingly small (Figure 2), making a kinetic resolution process of (±)-**1a** impractical. However, when the substrate was the scalemic (*R*)-**1a** formed by biocatalyzed oxidation of **1**, some improvement in the optical purity of the sulfoxide was obtained, although at the expense of the chemical yield, and performing the reaction in buffer pH 8.5 a maximum of 89% *ee* for (*R*)-**1a** was reached in the presence of 29% of **1b** (Figure S1). 

Interestingly, the two oxidation steps (**1** → **1a** → **1b**) are catalyzed by peroxygenase in a strictly consecutive manner, although the reaction rates of the individual steps appear quite similar, and as long as **1** is present in the reaction mixture, the enzyme does not transform **1a** into **1b**, suggesting a great difference in its affinity for the two substrates.

When sulfides **2** and **5** were used as substrates, the optical purity of the corresponding sulfoxides **2a** and **5a** remained nearly unchanged with increasing sulfone concentration, indicating even poorer enantiodiscrimination in the over-oxidation reaction. The optical purity of (*R*)-**4a**, on the other hand, increased in parallel with the sulfone formation, so that (*R*)-**4a** in almost doubled *ee* (57%) was obtained at 45% concentration of **4b** in the reaction mixture.

Although other plant peroxygenases have been reported to be active in the oxidation of *p*-substituted thioanisoles [55] and to two sulfur-containing pesticides [56,57], the investigated reactions were aimed at understanding the enzyme features rather than its application in organic synthesis and the optical purity of just one sulfoxide was determined [55]. Fungal peroxygenases (UPO and CPO) have been applied more extensively to sulfoxidation reactions and some differences can be highlighted for oat peroxygenase with respect to these enzymes. While CPO is mostly active on methyl sulfides, oat peroxygenase displays better acceptance for non-methyl groups on sulfur atom, as also reported for UPO. Although sulfoxide **1a** was obtained in higher optical purity by using UPO or CPO, the oxidation of **3** to **3a** is more selective with oat peroxygenase. 

The biocatalyzed formation of sulfones from sulfides seems to be specific for our peroxygenase-containing preparation, albeit not very selective, and could contribute to enhance the optical purity of target sulfoxides. 

## 3. Materials and Methods

### 3.1. General

Sulfides **1**, **3**–**5**, and aqueous *tert*-butyl hydroperoxide (70 wt% in H_2_O, 7.3 M) were obtained from Santa Cruz Biotechnology, Dallas, TX, USA. Sulfide **2**, sulfoxide (±)-**1a**, guaiacol and 2,2,5,7,8-pentamethyl-6-chromanol were purchased from Aldrich (Merck LifeScience, Milano, Italy). Horseradish peroxidase (53 purpurogallin units/mg) was from Sigma (Merck LifeScience s.r.l., Milan, Italy). Reference samples of the racemic sulfoxides (±)-**2a**–(±)-**5a** were obtained by standard oxidation of the sulfides with *m*-chloroperbenzoic acid in CH_2_Cl_2_. Whole oat seeds from organic crops (Ki Group, Torino, Italy) were bought at the local supermarket.

TLC analyses were performed on aluminum plates coated with silica gel and fluorescent indicator F254 revealing the compounds by UV. Column chromatography was performed on Silica gel 60 (Merck, 40–63 μm) using the specified eluents.

^1^H- and ^13^C-NMR spectra were recorded in CDCl_3_ on a Bruker AvanceTM 400 (Bruker Italia, Milano, Italy) spectrometer at 400.13 and 100.62 MHz, respectively. Optical rotations were measured on Jasco DIP-135 (Jasco Europe, Lecco, Italy) polarimeter using a 10 cm length cell.

### 3.2. Enzymatic Preparation from Oat Seeds

Lyophilized enzymatic preparation from oat seeds flour was obtained as previously reported [40]. Briefly, commercial whole seeds (60 g) of air-dried oat (*Avena sativa*) from organic crops were ground in a domestic blender and the obtained flour was defatted by washing with diethyl ether (3 × 150 mL), centrifuging at 4000 RPM (2930× *g*) and discarding the supernatant. The final residue was dried at room temperature overnight to give defatted flour (56 g).

Defatted oat flour was suspended in water (160 mL) and stirred for 10 min. The slurry was centrifuged at 4000 RPM for 7 min and the suspension was collected. The pellet was washed with water and the suspension centrifuged again. The pooled supernatant fractions were freeze-dried to give 5.2 g of a white light powder. For testing the activity, oat preparation (50 mg) was suspended in phosphate buffer pH 7.5 (5 mL) and methyl oleate (13 μL, 38 μmol) was added. After the addition of TBHP (6 μL, 44 μmol) the mixture was stirred at 25 °C and the reaction progress monitored by GC. An activity of 0.50 ± 0.05 μmol/mg/h was determined for the preparation used in this work.

### 3.3. HPLC Analyses

HPLC analyses were carried out on a Dionex instrument (ThermoFisher Scientific, Milano, Italy) equipped with an Ultimate 3000 high-pressure binary pump, an ASI-100 autosampler, a TCC-100 thermostated column compartment and a UVD-100 multiple wavelength detector set at 220, 230, 240 and 260 nm. Chromeleon software (version 6.7) was used for instrument control, data acquisition, and data handling. Chiral HPLC was performed on a Phenomenex (Phenomenex, Inc. Bologna, Italy) Lux 5 μm Cellulose-1 (250 × 4.6 mm) column eluting with *n*-hexane/EtOH 90:10 at flow 0.5 mL/min. The composition of reaction mixtures was determined at λ 220 nm by applying the suitable response factors for sulfoxides with respect to sulfides and for sulfones with respect to sulfoxides. Peak assignment was based on the reported elution order of enantiomers of sulfoxides on the same stationary phase [43,58]: *t_R_* 17.7 min [(*R*)-**1a**] and 19.4 min [(*S*)-**1a**]; 17.2 min [(*R*)-**2a**] and 19.0 min [(*S*)-**2a**]; 21.7 min [(*R*)-3a] and 25.9 min [(*S*)-**3a**]; 18.1 min [(*R*)-**4a**] and 19.7 min [(*S*)-**4a**]; 22.6 min [(*R*)-**5a**] and 25.3 min [(*S*)-**5a**].

### 3.4. Screening of Reaction Conditions for the Biocatalytic Oxidation of ***1***

To a suspension of **1** (22 mg, 0.18 mmol) in the solvent of choice (5 mL) the lyophilized enzymatic preparation (100 mg) was added and the resulting mixture was vigorously stirred at 25 °C (or other set temperature). The oxidant TBHP (15 μL, 0.11 mmol, 0.6 eqv.) was added in three portions over 1 h and the reaction course was monitored by chiral HPLC. At suitable times, aliquots (0.3 mL) of the reaction mixtures were withdrawn and extracted with diethyl ether (0.6 mL). The organic phase was separated, dried over anhydrous Na_2_SO_4_ and diluted with mobile phase before HPLC injection. 

### 3.5. Biocatalytic Oxidation of Sulfides ***1***–***5***

To a suspension of lyophilized enzymatic preparation (100 mg) and sulfides **1**–**5** (0.18 mmol) in phosphate buffer pH 7.5/acetone 8:2 *v*/*v* mixture (5 mL), TBHP (34 μL, 0.25 mmol, 1.4 eqv.) was added in three portions over 1 h and the reaction course was monitored by chiral HPLC, as described above. 

### 3.6. Enzymatic Synthesis of (R)-***1a***

The lyophilized enzymatic preparation (600 mg) was suspended in a 8:2 *v*/*v* 50 mM phosphate buffer pH 7.5:acetone mixture (30 mL) and **1** (280 mg, 2.26 mmol) was added. To the suspension, vigorously stirred at 25 °C, TBHP was added in three aliquots over 2 h (total volume 309 μL, 2.26 mmol, 1 eqv.) and the reaction progress was monitored by chiral HPLC. After 3 h the reaction mixture was extracted with 95:5 *v*/*v* EtOAc/MeOH (3 × 20 mL) centrifuging at 4000 RPM for 10 min to better separate the organic phase. The organic layers were pooled, dried over anhydrous Na_2_SO_4_ and taken to dryness to give pure (*R*)-**1a** as clear oil (304 mg, 2.17 mmol, 96% yield) in 80% *ee*; ^1^H- and ^13^C-NMR spectra were found in agreement with the literature data [58].

### 3.7. Improvement of the Optical Purity of (R)-***1a*** via Sulfone Formation

To a vigorously stirred suspension of **1** (280 mg, 2.26 mmol) and lyophilized enzymatic preparation (600 mg) in 50 mM phosphate buffer at pH 8.5 (30 mL) at 25 °C, TBHP was added in three aliquots over 3 h (total volume 463 μL, 3.39 mmol, 1.5 eqv.) and the reaction progress was monitored by chiral HPLC. After 4 h, when 29% of sulfone was formed, the reaction was extracted with 95:5 *v*/*v* EtOAc/MeOH (3 × 20 mL) and the organic layers were separated by centrifugation (4000 RPM for 10 min), collected and dried on anhydrous Na_2_SO_4_. The organic solvent was removed under reduced pressure and the residue purified by chromatography on Silica gel column eluting with *n*-hexane/EtOAc (from 70:30 to 30:70 *v*/*v*). Sulfone **1b** (92 mg, 0.59 mmol, 26% yield) was obtained as first eluting compound, followed by (*R*)-**1a** (203 mg, 1.45 mmol, 64% yield) with 89% *ee*; [α]^D^_25_ = +109 (*c* 1.2, acetone), lit. [α]^D^_25_ = +114 (*c* 1.5, acetone) for (*R*)-**1a** with 97% *ee* [58].

### 3.8. Enzymatic Synthesis of (S)-***3a***

The lyophilized enzymatic preparation (600 mg) was suspended in a 50 mM phosphate buffer at pH 7.5 (30 mL) and sulfide **3** (231 mg, 1.50 mmol) was added. To the suspension, vigorously stirred at 25 °C, TBHP was added in three aliquots over 2 h (total volume 206 μL, 1.50 mmol, 1 eqv.) and the reaction progress was monitored by chiral HPLC analysis. After 3 h the reaction mixture was extracted with 95:5 *v*/*v* EtOAc/MeOH (3 × 20 mL) and the organic layers were separated by centrifugation (4000 RPM for 10 min), dried over anhydrous Na_2_SO_4_ and taken to dryness to give pure (*S*)-**3a** as a clear oil (230 mg, 1.35 mmol, 90% yield) in 92% *ee*; [α]^D^_25_ = +195.3 (*c* 1.2, CHCl_3_), lit. [α]^D^_25_ = +191.4 (*c* 1.2, CHCl_3_) for (*S*)-**3a** 86% *ee* [58]. ^1^H- and ^13^C-NMR spectra were found in agreement with the literature data [58].

### 3.9. Assay for Peroxidase Activity

Lyophilized enzymatic preparation (50 mg) was suspended in a 50 mM phosphate buffer pH 7.5 (2.5 mL) in a cuvette and a 96 mM water solution of guaiacol (0.25 mL, 0.024 mmol) was added. The volume was adjusted to 3 mL with 50 mM phosphate buffer pH 7.5 and the reaction was started by the addition of 3% *w*/*w* H_2_O_2_ solution (25 μL, 0.024 mmol). The reaction progress was monitored spectrophotometrically by the increase in absorption at λ 470 nm for the product tetraguaiacol (ε = 26.6 mM^−1^ cm^−1^) over 3 min. Pure horseradish peroxidase was used as positive control. 

### 3.10. Isolation of Microsome Fraction from Oat Seeds

The lyophilized enzymatic preparation (100 mg) was suspended in a 50 mM phosphate buffer pH 7.5 (10 mL), stirred for 10 min at 25 °C and centrifuged at 10,000× *g* for 10 min. The obtained pellet was discarded and the retained supernatant was further centrifuged at 100,000× *g* for 45 min by an ultracentrifuge. After decanting the supernatant, the microsome pellet was washed by resuspension in 50 mM phosphate buffer pH 7.5 and recentrifugation as above. The final pellet was suspendend in 50 mM phosphate buffer pH 7.5 (5 mL) and sulfide **1** (6 mg, 0.05 mmol) was added. The reaction was started by adding TBHP (7 μL, 0.05 mmol) at 25 °C under magnetic stirring and monitored by HPLC. After 1 h, a 33% conversion of **1** was measured and (R)-**2** was formed in 76% *ee*. In a parallel reaction by using the whole lyophilized enzymatic preparation in the same reaction conditions, sulfide **1** was fully converted in 30 min and (R)-**2** in 75% *ee* was obtained.

## 4. Conclusions

A raw peroxygenase-containing preparation was used as a biocatalyst in the asymmetric oxidation of sulfides to optically active sulfoxides and high reaction rate, coupled with selectivity ranging from moderate to excellent, was observed for five different substrates. The formation of sulfones as over-oxidation products was also detected in the biocatalyzed reaction and investigated in detail.

Oat peroxygenase tolerated different reaction conditions without significant changes in its activity, confirming to be a robust enzyme also suitable for reactions in preparative scale for its low cost and easy availability. The activity in sulfoxidation reactions described here, in addition to that reported in the epoxidation of different substrates, makes this plant peroxygenase a promising and useful tool in organic synthesis.

## Figures and Tables

**Table 1:** Biocatalyzed oxidation of sulfides with oat peroxygenase *^a^*.

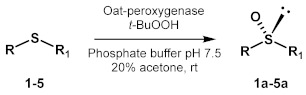					
Compound	R	R_1_	Sulfoxide (%) *^b^*	Ee (%) *^c^*	Configuration *^d^*
**1**	Ph	CH_3_	93	80	*R*
**2**	Ph	CH_2_CH_3_	60	58	*R*
**3**	Ph	CH_2_OCH_3_	60	90	*S ^e^*
**4**	*p*-CH_3_-Ph	CH_3_	75	34	*R*
**5**	Bn	CH_3_	>99	28	*R*

*^a^* Experimental conditions: peroxygenase preparation (100 mg), substrate (0.18 mmol), *t*-BuOOH (0.25 mmol), phosphate buffer pH 7.5/acetone 8:2 (5 mL), 25 °C; *^b^* at 1 h reaction time, no sulfones were present in the reaction mixtures; *^c^* determined by chiral HPLC analysis; *^d^* assigned by elution order of the major enantiomer; *^e^* absolute configuration is reversed due to a change in the substituent priority according to the sequence rules.

## Data Availability

The data presented in this study are available in this manuscript.

## Supplementary Materials

The following supporting information can be downloaded at: https://www.mdpi.com/article/10.3390/ijms24087464/s1.
