# Supplementary material for: Expanding the Use of Peroxygenase from Oat Flour in Organic Synthesis: Enantioselective Oxidation of Sulfides
Source: Int J Mol Sci. 2023 Apr 18;24(8):7464. doi: 10.3390/ijms24087464 (PMC10138840; doi:10.3390/ijms24087464)
Supplement: Supplementary file 1 [file ijms-24-07464-s001.zip › ijms-2348143-supplementary.pdf]

Expanding the use of peroxygenase from oat flour in organic synthesis: enantioselective oxidation of sulfides

Claudia Sanfilippo, Federica Cernuto and Angela Patti*

CNR - Istituto di Chimica Biomolecolare, Via Paolo Gaifami 18, I-95126 Catania, Italy

SUPPORTING INFORMATION

*Corresponding author. E-mail: angela.patti@cnr.it

HPLC chromatograms of reference racemic sulfoxides and chiral sulfoxides obtained by biocatalyzed oxidation of sulfides — Pages SI-1 – SI-5

Figure S1. Variation of the enantiomeric excess of sulfoxide **1a** at different concentration of sulfone **1b** — Page SI-6

Figure S2. ^{1}H- and ^{13}C-NMR spectra of sulfoxide **1a** — Page SI-7

Figure S3. ^{1}H- and ^{13}C-NMR spectra of sulfoxide **3a** — Page SI-8

HPLC analyses

Chiral HPLC analyses were performed on a Phenomenex Lux 5μm Cellulose-1 (250 x 4.6 mm) column eluting with *n*-hexane/EtOH 90:10 at flow 0.5 mL/min. (±)-**1a** was purchased from Aldrich while other racemic samples of sulfoxides were obtained by standard oxidation of the parent sulfides with *m*-Cl-PhCOOOH in CH_2Cl_2.

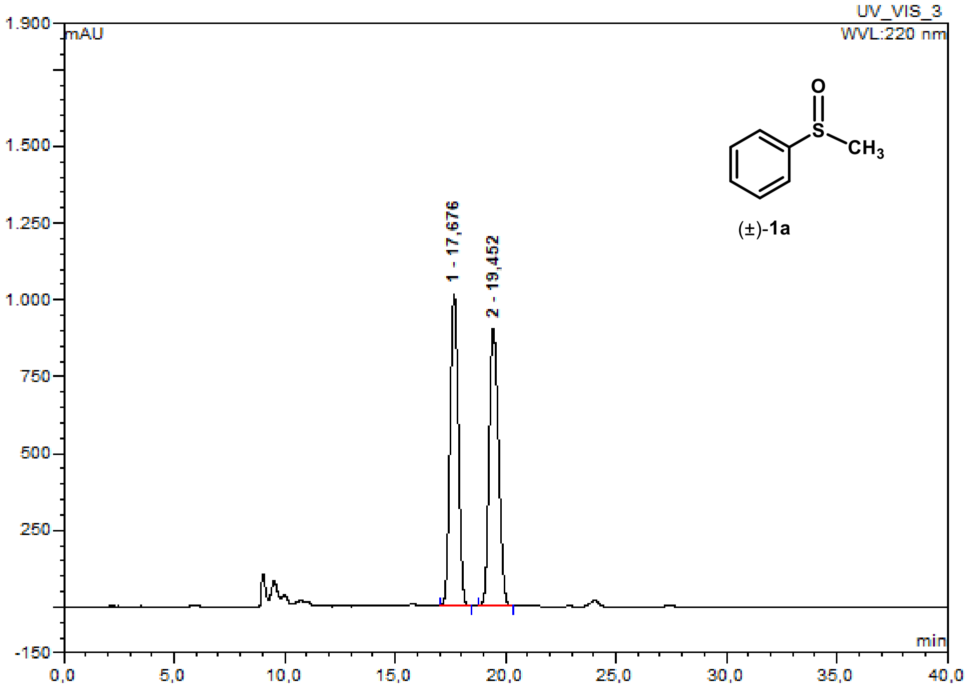

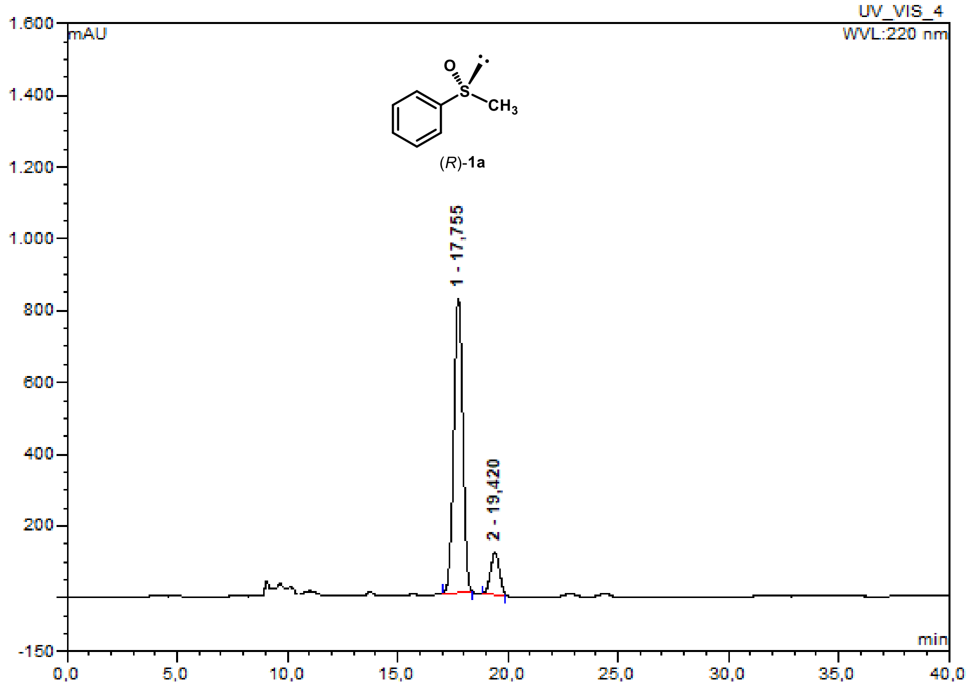

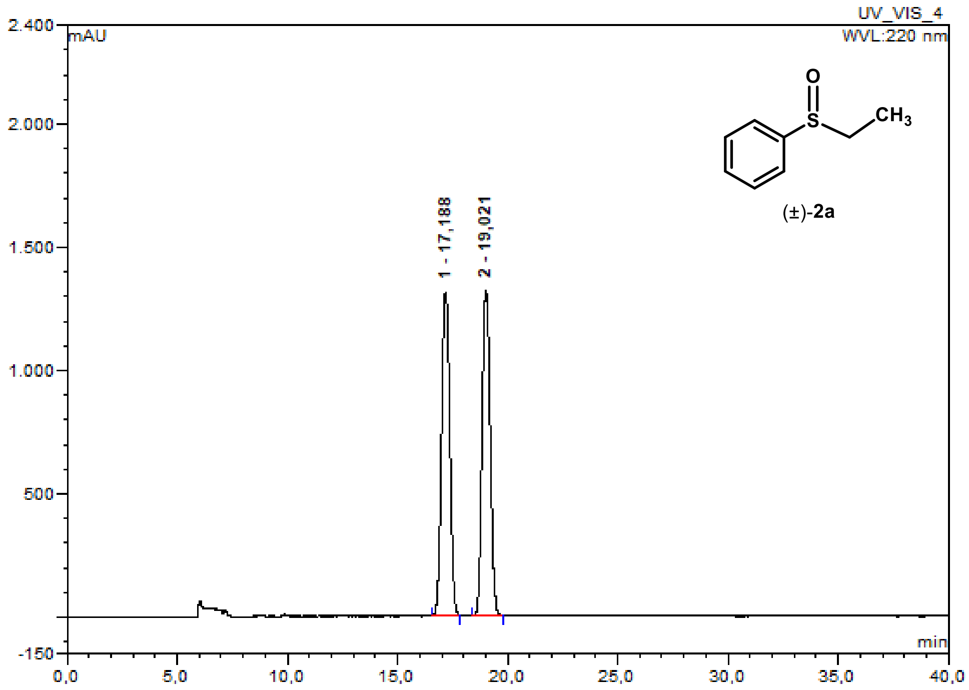

UV_VIS_4
WVL:220 nm
mAU
2.400
2.000
1.500
1.000
500
-150
1 - 17,188
2 - 19,021
CH3
(±)-2a
min
0,0
5,0
10,0
15,0
20,0
25,0
30,0
35,0
40,0

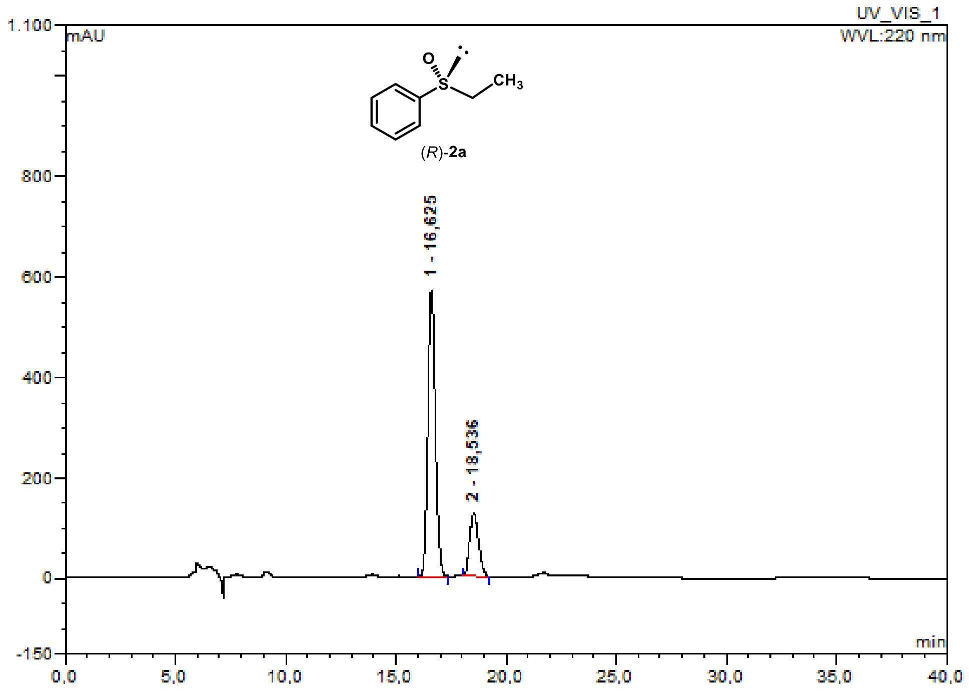

UV_VIS_1
WVL:220 nm
mAU
1.100
800
600
400
200
0
-150
CH3
(R)-2a
1 - 16,625
2 - 18,536
min
0,0
5,0
10,0
15,0
20,0
25,0
30,0
35,0
40,0

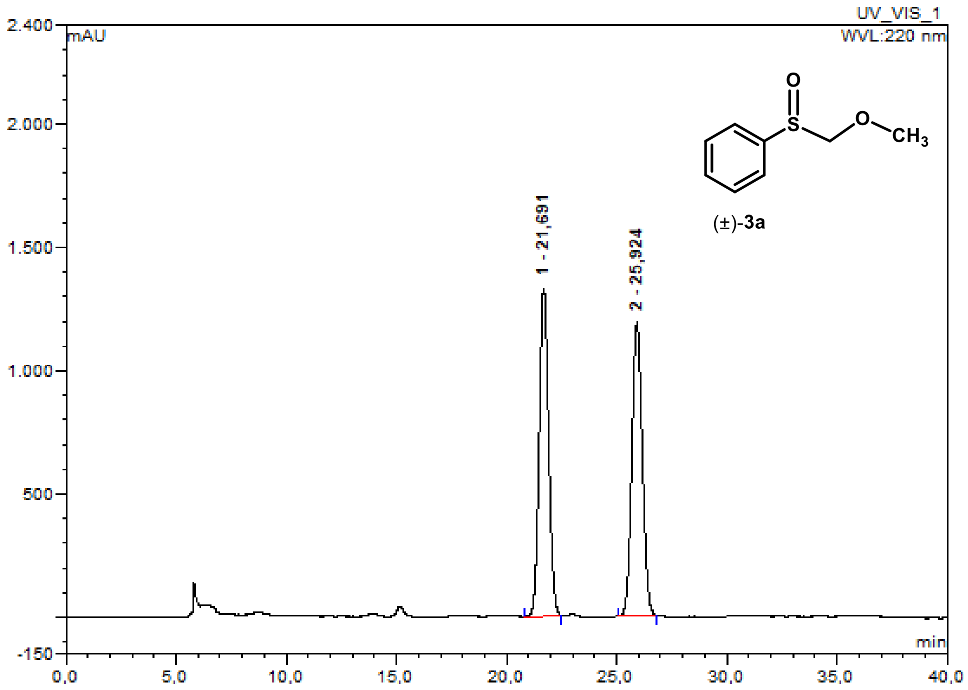
UV_VIS_1
WVL:220 nm
mAU
2.400
2.000
1.500
1.000
500
-150
1 - 21,691
2 - 25,924
(±)-3a
min
0,0
5,0
10,0
15,0
20,0
25,0
30,0
35,0
40,0

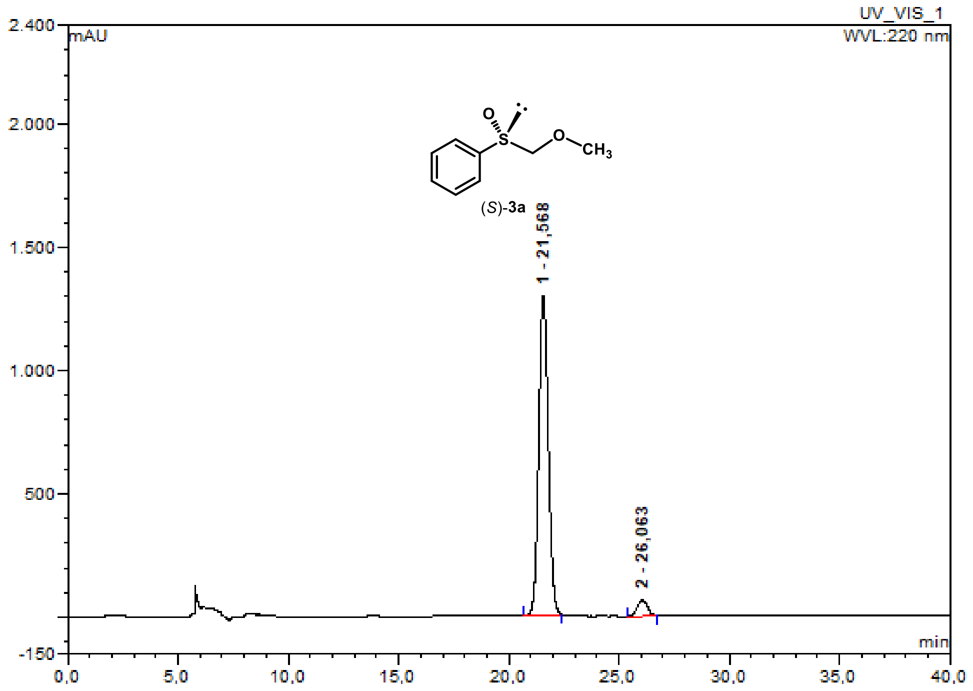
UV_VIS_1
WVL:220 nm
mAU
2.400
2.000
1.500
1.000
500
-150
(S)-3a
1 - 21,568
2 - 26,063
min
0,0
5,0
10,0
15,0
20,0
25,0
30,0
35,0
40,0

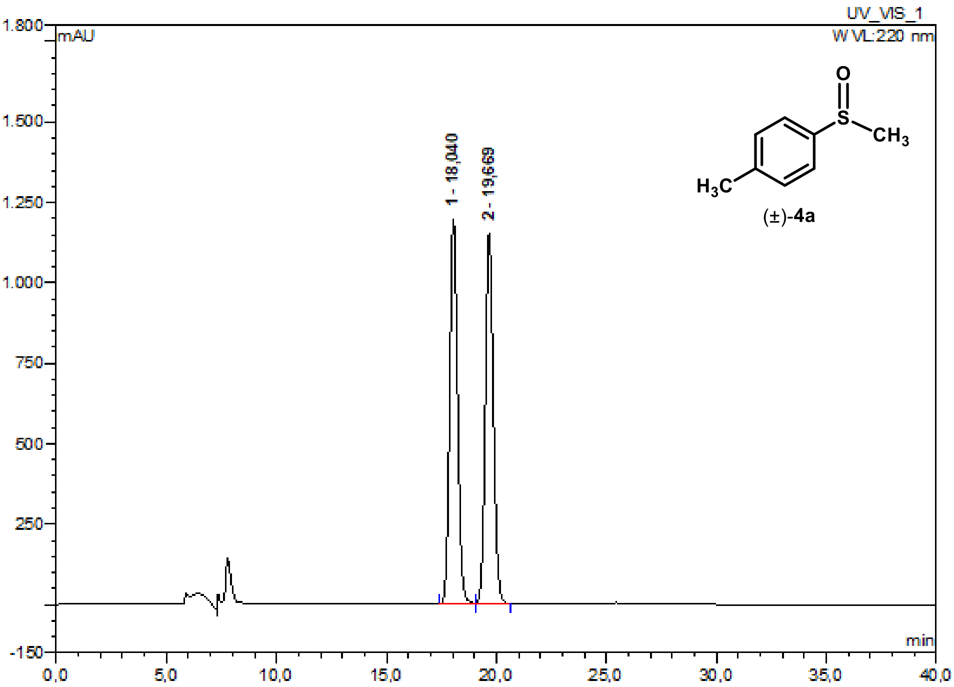
UV_VIS_1
WVL:220 nm
mAU
1 - 18,040
2 - 19,669
H3C
CH3
O
S
(±)-4a
min

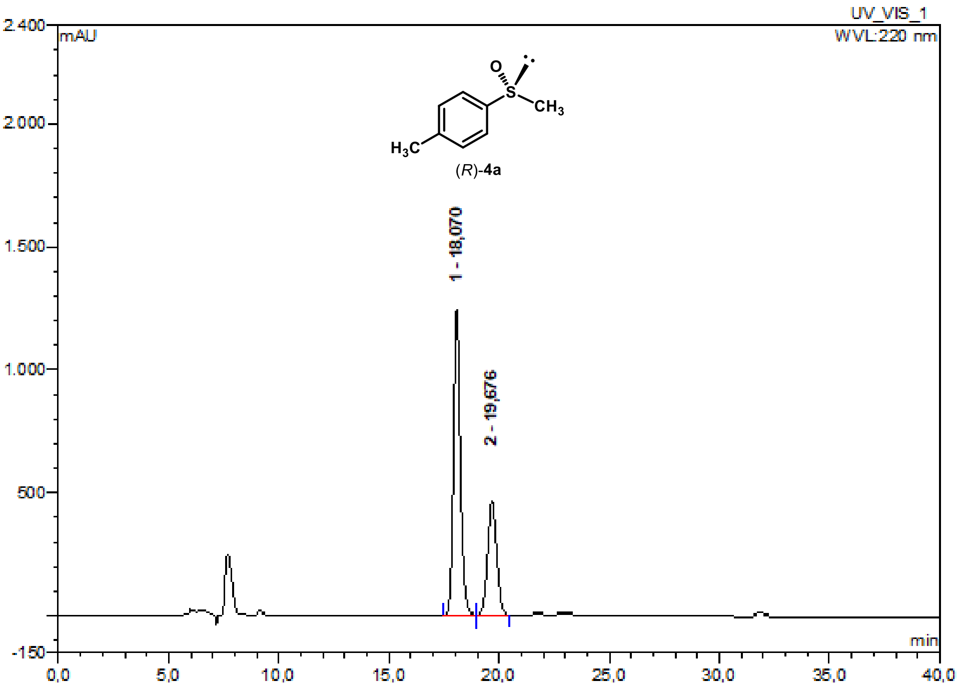
UV_VIS_1
WVL:220 nm
mAU
O
S
CH3
H3C
(R)-4a
1 - 18,070
2 - 19,676
min

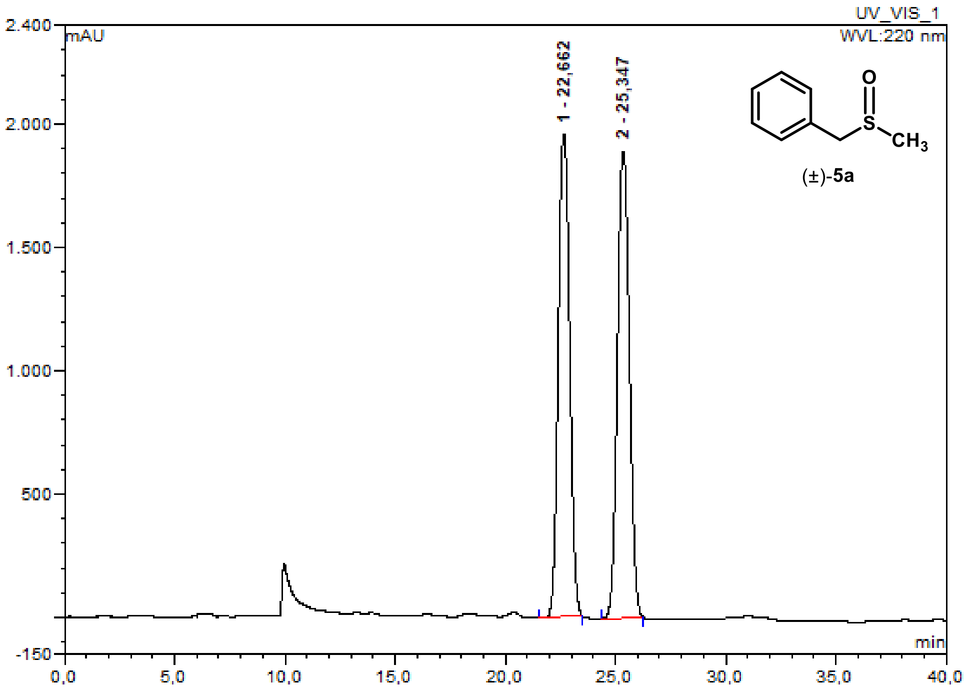
UV_VIS_1
WVL:220 nm
mAU
1 - 22,662
2 - 25,347
(±)-5a
min

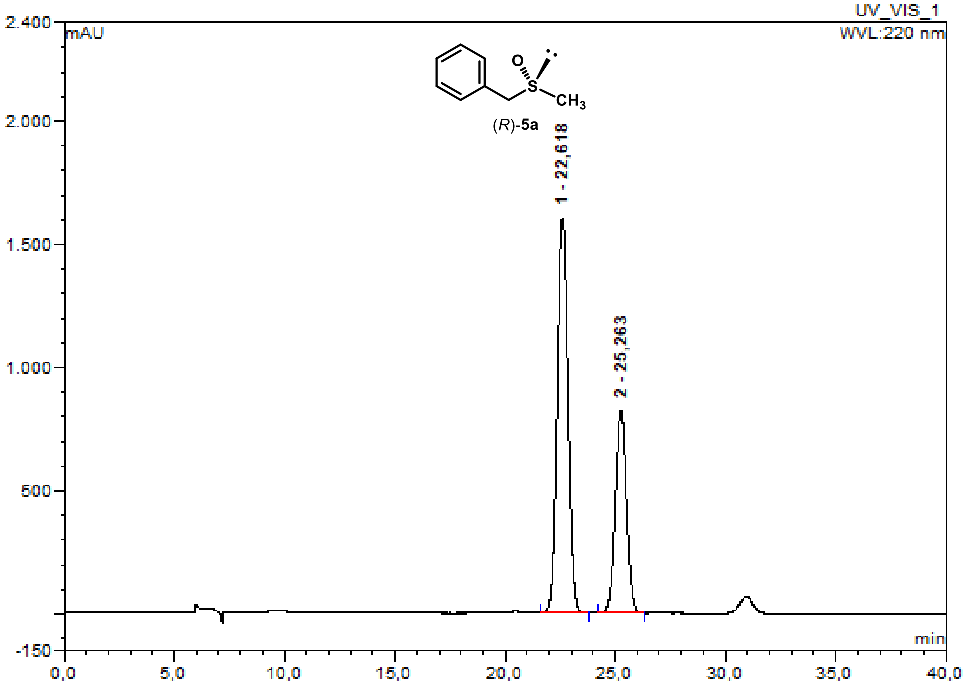
UV_VIS_1
WVL:220 nm
mAU
(R)-5a
1 - 22,618
2 - 25,263
min

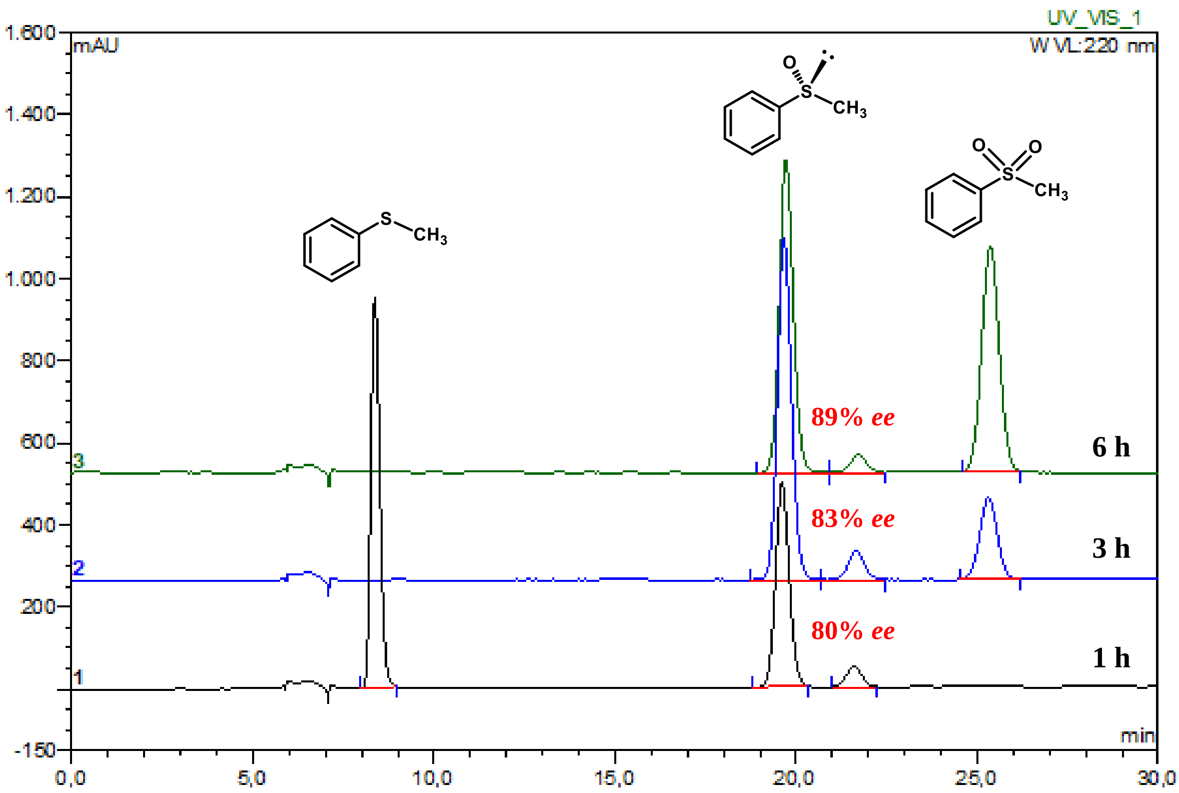

Figure S1. Variation of the enantiomeric excess of sulfoxide **1a** at different concentration of sulfone **1b**

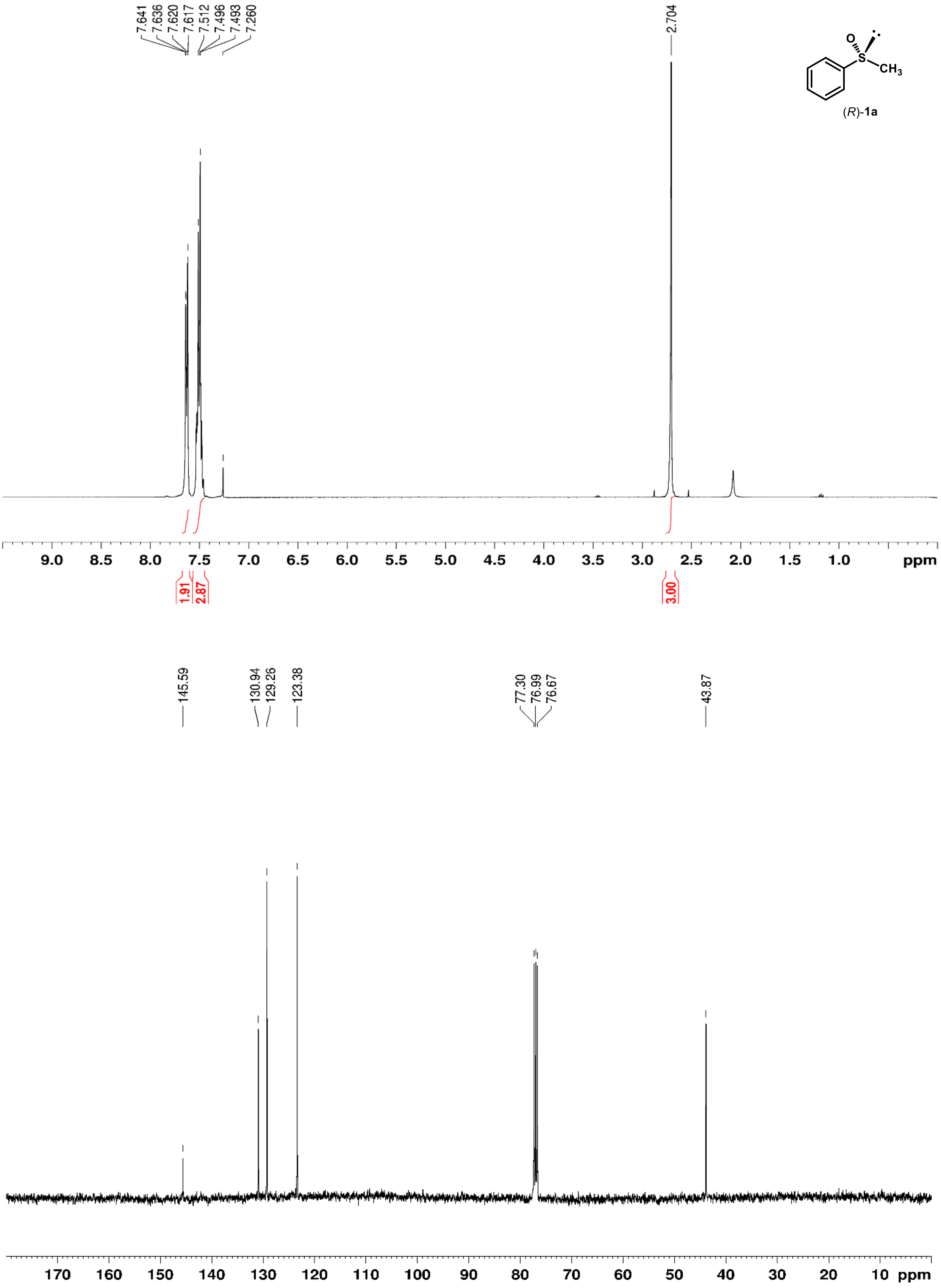

Figure S2. ^{1}H- and ^{13}C-NMR spectra of sulfoxide **1a**

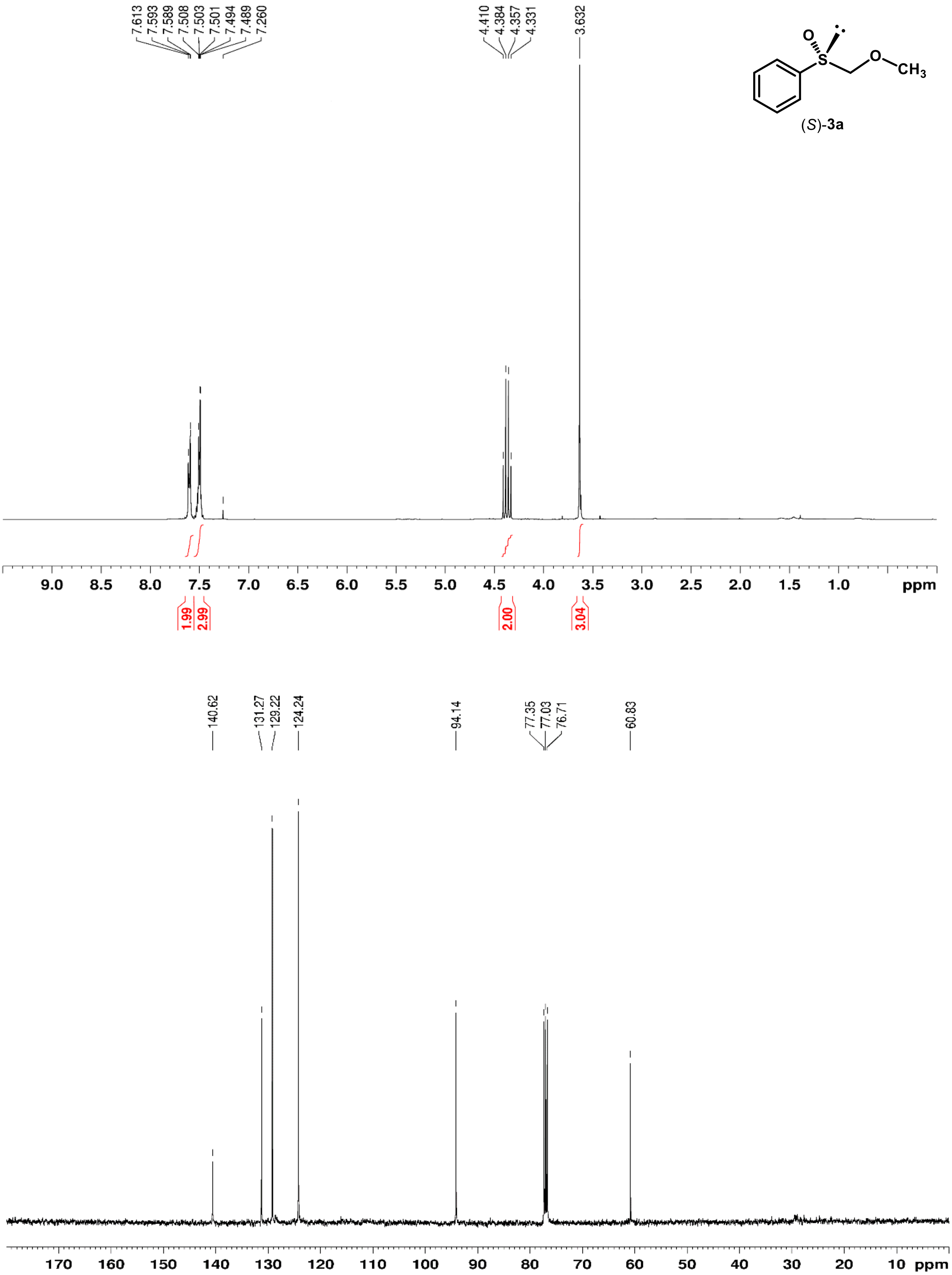

Figure S3. ^{1}H- and ^{13}C-NMR spectra of sulfoxide **3a**
